# Prognostic significance of pan-immune-inflammatory value in adverse cardiovascular and cerebrovascular events post-percutaneous coronary intervention in diabetic patients with coronary heart disease

**DOI:** 10.3389/fcvm.2026.1750706

**Published:** 2026-07-07

**Authors:** Qinghong Liu, Mao Tian, Jiangjun Guo, Wenhao Chen, Wenting Wu, Huadong Yu, Bo Zhu

**Affiliations:** 1 Department of Cardiology, Luzhou People’s Hospital, Luzhou, Sichuan, China; 2 Department of Cardiology, The Fifth People's Hospital of Yibin, Yibin, Sichuan, China; 3 Department of Cardiology, Fushun County Traditional Chinese Medicine, Zigong, Sichuan, China; 4 Department of Cardiology, Anyue Hospital of Traditional Chinese Medicine, Anyue, Sichuan, China; 5 Department of Cardiology, Longchang People’s Hospital, Longchang, Sichuan, China; 6 Department of Cardiology, The Affiliated Hospital of Southwest Medical University, Luzhou, Sichuan, China

**Keywords:** coronary heart disease, diabetes mellitus, major adverse cardiovascular and cerebrovascular events, pan-immune-inflammatory value, percutaneous coronary intervention

## Abstract

**Background:**

The pan-immune-inflammation value (PIV) is a novel biomarker reflecting systemic inflammation. Its role in predicting adverse cardiovascular and cerebrovascular events in diabetic patients after percutaneous coronary intervention (PCI) is unclear.

**Objectives:**

This study evaluated PIV's prognostic value for major adverse cardiovascular and cerebrovascular events (MACCE) post-PCI in diabetics with coronary heart disease (CHD), and compared it to other inflammation-based markers like systemic immune-inflammation index (SII), neutrophil-to-lymphocyte ratio (NLR), and platelet-to-lymphocyte ratio (PLR).

**Methods:**

Retrospective analysis of diabetic CHD patients undergoing PCI. PIV was calculated as (neutrophil × platelet × monocyte)/lymphocyte counts from pre-procedural blood. Optimal cutoff determined via ROC curve. Patients stratified into high/low PIV groups. Follow-up for MACCE (e.g., MI, stroke, revascularization). Kaplan–Meier (KM) survival, Cox regression, and ROC comparisons assessed outcomes.

**Results:**

Over 24-month median follow-up, 52 MACCE occurred (24.8%). High-PIV group had higher incidence (37.7% vs. 11.5%, *p* < 0.001) and worse MACCE-free survival (log-rank *p* < 0.001). Multivariate Cox confirmed high PIV as independent predictor (adjusted HR = 2.87, 95% CI: 1.55–5.32, *p* = 0.001). PIV AUC = 0.74 (95% CI: 0.68–0.80), outperforming SII (0.69), NLR (0.66), and PLR (0.64; DeLong's test *p* < 0.05 vs. NLR/PLR).

**Conclusions:**

PIV is a robust, independent predictor of MACCE post-PCI in diabetics, with superior accuracy over other markers. It offers cost-effective risk stratification. Limitations: retrospective design; prospective validation needed.

## Highlights

1. Pan-immune-inflammatory value reflects systemic inflammatory and immune imbalance in diabetic coronary heart disease patients.
2. Elevated pre-procedural PIV is associated with increased risk of adverse cardiovascular and cerebrovascular events after PCI.
3. PIV demonstrated superior prognostic performance compared with conventional inflammatory indices.
4. High PIV was associated with poorer long-term MACCE-free survival following coronary intervention.
5. PIV may serve as a simple and cost-effective biomarker for individualized risk stratification in diabetic patients undergoing PCI.

## 1 Introduction

Coronary heart disease (CHD) remains a leading cause of morbidity and mortality worldwide, with its incidence steadily rising across both high-income and low-to-middle-income countries, thereby globally exacerbating the overall burden on public health systems (1). Additionally, the prevalence of diabetes mellitus (DM) remains alarmingly high, and its pervasive impact on cardiovascular risk is well-documented, in which diabetics are not only more prone to developing extensive and aggressive coronary atherosclerosis, but also experience noticeably worse prognoses post-revascularization interventions (2). Although remarkable strides have been made in refining percutaneous coronary intervention (PCI) techniques and optimizing guideline-directed medical therapies, Despite advances in percutaneous coronary intervention (PCI) techniques and guideline-directed medical therapy, patients remain at risk of major adverse cardiovascular and cerebrovascular events (MACCE) following PCI. This risk is particularly elevated among patients with diabetes mellitus, who frequently present with more complex coronary artery disease and a higher burden of cardiovascular risk factors. Consequently, improved risk stratification tools are needed to identify patients at increased risk of adverse outcomes after PCI (2, 3).

A substantial body of evidence has established that inflammation and immune system activation play a central role in the pathogenesis of both cardiovascular and cerebrovascular complications. Atherosclerosis is now recognized as a chronic inflammatory disease of the arterial wall, involving endothelial dysfunction, monocyte/macrophage infiltration, neutrophil activation, platelet-leukocyte interactions and adaptive immune dysregulation (4). In the diabetic milieu, insulin resistance, hyperglycemia and dyslipidemia promote low-grade systemic inflammation, oxidative stress and vascular injury, thereby magnifying the risk of adverse events after PCI (5).

Given this mechanistic linkage, inflammatory and immune biomarkers may provide additional prognostic insight beyond traditional risk factors in CHD patients post-PCI particularly in those with diabetes.

The pan-immune-inflammatory value (PIV) reflects the balance between innate pro-inflammatory/pro-thrombotic components (neutrophils, monocytes, platelets) and the adaptive immune component (lymphocytes).

PIV provides a comprehensive reflection of systemic immune-inflammatory status by integrating neutrophil, monocyte, platelet, and lymphocyte counts into a single biomarker (6). Initially developed in oncology, elevated PIV has been associated with poor prognosis and survival outcomes across multiple malignancies (7, 8). More recently, cardiovascular studies have demonstrated that higher PIV levels are associated with increased coronary artery disease severity, impaired coronary collateral circulation, and adverse cardiovascular outcomes, including major adverse cardiac events (9, 10).

Despite increasing interest in PIV within cardiovascular medicine, evidence regarding its prognostic value in diabetic patients with CHD undergoing PCI remains limited. Diabetic patients represent a high-risk population characterized by persistent inflammation, endothelial dysfunction, and increased susceptibility to adverse post-PCI outcomes. However, most previous studies evaluated mixed cardiovascular populations and focused primarily on major adverse cardiac events without comprehensively assessing cerebrovascular complications such as ischemic stroke or transient ischemic attack.

Therefore, the present study aimed to investigate the prognostic significance of pre-procedural PIV for predicting major adverse cardiovascular and cerebrovascular events in diabetic patients with CHD undergoing PCI and to compare its predictive performance with other established inflammatory biomarkers.

## 2 Materials and methods

### 2.1 Study population

The current retrospective observational investigation involved a cohort of 210 consecutive diabetics with CHD who scheduled undergoing PCI at Luzhou Municipal People's Hospital between May 2023 and December 2023. All PCI procedures were implemented in accordance with the established guidelines for coronary revascularization as outlined by the European Society of Cardiology (ESC) and the American College of Cardiology (ACC). Inclusion criteria required that cases be adults (≥18 years) with angiographically confirmed CHD, characterized by the presence of at least one coronary artery exhibiting a ≥50% stenotic lesion. DM diagnosis was established using the criteria set forth by the American Diabetes Association (ADA), involving fasting plasma glucose surpassing 7.0 mmol/L, HbA1c exceeding 6.5%, or the current usage of antidiabetic pharmacotherapy. Each case underwent successful PCI with stent implantation, and the availability of comprehensive baseline clinical and laboratory data, particularly prior to the procedure, was confirmed.

Exclusion criteria comprised: (1) presence of active infection, autoimmune or hematologic disorders, malignancy, or any chronic inflammatory disease; (2) severe hepatic or renal impairment, defined as an estimated glomerular filtration rate (eGFR) below 30 mL/min/1.73 m^2^; (3) a history of coronary artery bypass grafting (CABG); (4) recent major surgical or traumatic events within the preceding three months; (5) chronic administration of corticosteroids or immunosuppressants; and (6) incomplete follow-up or missing critical laboratory data.

Post-inclusion, we categorized cases into low-PIV and high-PIV groups on the basis of optimal threshold for the PIV, which was indicated through receiver operating characteristic (ROC) curve analysis utilizing the Youden index. Baseline demographic, clinical, and angiographic information was precisely extracted from electronic medical records, and the accuracy of these data was independently verified by two trained investigators to ensure consistency and reliability.

The research was strictly implemented to the principles compiled in the Declaration of Helsinki. Both informed consent and the protocol were obtained by the Ethics Committee of Luzhou Municipal People's Hospital.

### 2.2 Data collection and laboratory measurements

It was attempted to extract baseline demographic, clinical, angiographic, and laboratory information from the hospital's electronic medical record system at the time of patient admission. The laboratory analyses involved a comprehensive array of tests, comprising complete blood count, renal and hepatic function panels, lipid profiling, and HbA1c quantification. Fasting venous blood samples were collected within 24 h before PCI and analyzed in the hospital central laboratory.

The PIV's computation was formulated in the following:

$$\mathrm{PIV}=\frac{(\mathrm{neutrophil}\;\mathrm{count}\times \mathrm{monocyte}\;\mathrm{count}\times \mathrm{platelet}\;\mathrm{count})}{\mathrm{lymphocyte}\;\mathrm{count}}$$

Cell counts were expressed in units of ×10^9^/L. To implement comparative analysis, additional indices indicative of inflammation were computed, involving the systemic immune-inflammation index (SII), defined as the product of platelet count and neutrophil count divided by lymphocyte count, as well as the neutrophil-to-lymphocyte ratio (NLR) and platelet-to-lymphocyte ratio (PLR).

The ROC curve analysis was implemented to ascertain the optimal PIV cut-off for predicting MACCE was determined using ROC curve analysis. Utilizing the Youden index, it was feasible to pinpoint the threshold that maximized the sum of sensitivity and specificity, promoting the classification of cases into two principal groups for subsequent statistical examinations.

### 2.3 Definition of clinical outcomes and follow-up

MACCE was defined as a composite endpoint including cardiovascular death, non-fatal myocardial infarction, ischemic stroke/transient ischemic attack, and repeat target-vessel revascularization. Definitions were applied consistently throughout all analyses. MACCE was conceptualized as a composite outcome comprising: (1) cardiovascular mortality, (2) non-fatal MI, (3) ischemic stroke or TIA, and (4) repeat target-vessel revascularization, all of which were confirmed through coronary angiographic examination. Cardiovascular mortality was defined as death attributable to acute myocardial infarction, decompensated heart failure, sudden cardiac arrest, or fatal arrhythmic events. Non-fatal MI was diagnosed on the basis of the Fourth Universal Definition of Myocardial Infarction, requiring a concomitant elevation in cardiac troponin levels, in the presence of ischemic symptoms or newly acquired electrocardiographic and/or imaging evidence of myocardial infarction. Diagnosis of ischemic stroke and TIA was made by neurologists based on clinical symptomatology and brain imaging modalities, specifically CT/MRI. Repeat revascularization was classified as the need for any subsequent PCI or CABG conducted on the target vessel due to CAD's restenosis or progression.

Follow-up evaluations were implemented systematically at 1, 6, 12, and 24 months following PCI, utilizing outpatient consultations or structured telephonic interviews, both of which were undertaken by trained personnel who were blinded to the patient's PIV groupings. The median follow-up duration across the entire cohort was approximately two years, which provided sufficient temporal coverage to capture both early and late-stage adverse cardiovascular and cerebrovascular events. The occurrence and timing of these events were precisely validated through the examination of hospital records, procedural documentation, and discharge summaries.

Patients lost to follow-up or those who opted to withdraw consent were censored at the point of their last recorded contact. Mortality data were cross-verified through linkage with the national death registry where such data were accessible. The follow-up completion rate was robust, exceeding 95%.

These methods enabled comprehensive assessment of cardiovascular and cerebrovascular outcomes., promoting a precise and nuanced assessment of the prognostic value of the PIV as a biomarker for predicting long-term post-PCI complications in diabetics with CAD.

### 2.4 Survival analysis

To comprehensively figure out the PIV's long-term prognostic impact, survival outcomes underwent precise analysis utilizing the Kaplan–Meier (KM) method. The primary endpoint, Major adverse cardiovascular and cerebrovascular event (MACCE)-free survival, was defined as the duration from the date of PCI to the earliest occurrence of a MACCE or to the date of the last follow-up visit, whichever occurred first. KM survival curves were plotted to depict the cumulative probability of remaining free from MACCE in the both principal groups. The log-rank test was employed to compare survival distributions between the two principal groups, promoting an assessment of differences in event-free survival on the basis of PIV classification. Cases who remained alive and event-free at the end of the follow-up period were censored at their last recorded contact date, hindering potential bias from ongoing follow-up.

### 2.5 Cox regression (CR) analysis

To identify MACCE's independent predictors during the follow-up period, we employed Cox proportional hazards regression (CPHR) models. The time-to-event variable was defined as the interval between the date of PCI and the first occurrence of MACCE or the ending of the follow-up period, whichever occurred first. Initial univariate CR analyses were implemented to find out the link of each baseline variable with the incidence of MACCE. Variables with a *p*-value below 0.10 in the univariate analyses were subsequently incorporated into a multivariate CR model through a forward stepwise selection method, promoting the adjustment of potential confounders and the identification of the most relevant predictors while minimizing model complexity. In the final multivariate model, clinically significant covariates, comprising age, sex, presence of hypertension, levels of HbA1c, eGFR, and the extent of CAD, were retained as potential independent predictors, given their established relevance in cardiovascular risk stratification. The proportional hazards assumption was assessed using Schoenfeld residuals.

To quantify the strength of the links of the identified predictors with the risk of MACCE, HRs with corresponding 95% confidence intervals (CIs) were calculated, providing a precise estimation of the relative risks associated with each predictor while accounting for potential interactions and confounding effects. This approach provided a robust and detailed evaluation of the factors influencing long-term cardiovascular and cerebrovascular outcomes in the study population.

### 2.6 Subgroup and sensitivity analyses

To rigorously assess the robustness of the prognostic utility of the PIV in predicting MACCE, comprehensive subgroup analyses were implemented through stratified CPHR models. Prespecified subgroups were delineated on the basis of fundamental clinical determinants, involving glycemic control status (HbA1c ≤7% vs. >7%), insulin therapy (users vs. non-users), and the extent of CAD (single-vessel vs. multivessel disease). For each subgroup, HRs plus 95% CIs were computed to quantify the link of PIV category (high vs. low) with the risk of MACCE.

To figure our potential effect modification and ascertain whether the influence of PIV on MACCE risk was consistent across subgroups, interaction terms between PIV and each stratification variable were systematically incorporated into the CR framework. This approach allowed for a detailed examination of whether PIV's prognostic impact was modified by metabolic control, therapeutic regimen, or the anatomical complexity of CAD. The outcomes of these stratified analyses were visually summarized using forest plots, which provided a graphical representation of the HRs and 95% CIs for each subgroup. This realized a clear comparison of the consistency and magnitude of the PIV effect across distinct patient subgroups. These additional analyses were implemented to ensure that the predictive capacity of PIV remained stable and robust across a range of metabolic, therapeutic, and anatomical contexts in diabetics with CAD post-PCI.

### 2.7 Comparative predictive analysis

To precisely compare the PIV's prognostic performance with other established inflammation-based indices, an integrated approach encompassing ROC curve analyses and CPHR models was employed. The indices selected for comparison involved SII, NLR, and PLR, all of which have been proposed as potential biomarkers of systemic inflammation with relevance to cardiovascular outcomes.

For each biomarker, the area under the ROC curve (AUC) was computed as a measure of discriminative ability to forecast the occurrence of MACCE in the post-PCI setting. Differences in AUC values between PIV and the other inflammation-based indices were examined using DeLong's test, promoting the comparison of the AUCs of correlated ROC curves and determining the statistical significance of any observed differences in predictive accuracy.

To further refine comprehending of the independent links of these inflammatory markers with clinical outcomes, multivariate CPHR models were developed, incorporating PIV, SII, NLR, and PLR as covariates. These models were adjusted for a comprehensive array of potential confounders, comprising demographic factors (age, sex), clinical variables (hypertension, glycemic control), renal function (e.g., estimated glomerular filtration rate), and the extent of coronary artery disease (e.g., number of affected vessels), all of which are well-established determinants of cardiovascular risk.

The comparative performance of these biomarkers was summarized in a detailed tabular format, with relative HRs plus 95% CIs presented for each inflammation-based index. This allowed for a robust evaluation of the independent prognostic value of each marker in forecasting MACCE incidence.

### 2.8 Statistical analysis

Statistical analyses were performed using SPSS and R software. Continuous variables' assessment was initially undertaken for adherence to a normal distribution through the Kolmogorov–Smirnov test. For variables with a normal distribution, data description was through mean plus/minus standard deviation (SD), and comparable analysis of between-group differences was carried out via the independent-samples *t*-test. For abnormally distributed variables, it was attempted to express data in form of medians along with interquartile ranges (IQR), and comparable analysis of groups was carried out via the Mann–Whitney *U*-test. Categorical variables' expression was through frequency/percentage, and comparable analysis of differences particularly between two principal groups was undertaken through either the Chi-square test or Fisher's exact test.

The prognostic value of the PIV and other inflammation-based indices (SII, NLR, and PLR) was figured out both as continuous and categorical variables. Utilizing ROC curve analysis, The optimal PIV cut-off for predicting MACCE was determined using ROC curve analysis, with the Youden index employed to detect the cut-off point that maximized both sensitivity and specificity. To find out the discriminatory power of the different inflammatory indices, pairwise ROC curve comparisons were implemented via DeLong's test. KM survival analysis was applied to estimate cumulative event-free survival rates, and significant differences in survival curves particularly between groups underwent analysis through the log-rank test.

To identify MACCE's independent predictors, CPHR models were developed. Variables demonstrating statistical significance (*p* < 0.10), particularly in univariate analyses, were subsequently involved in the multivariate regression model via a forward stepwise selection procedure. The proportional hazards assumption for the Cox model was precisely tested via Schoenfeld residuals, ensuring the validity of the model's assumptions. Furthermore, it was attempted to implement subgroup analyses through stratified Cox models utilizing clinically relevant factors, comprising HbA1c level, insulin therapy usage, and the extent of CAD (single-vessel vs. multivessel). Interaction terms between PIV and each subgroup factor were incorporated into the models to test for potential effect modification.

A two-tailed approach was employed to conduct statistical analysis, and denoting statistical significance was through P falling below 0.05.

## 3 Results

### 3.1 Baseline characteristics

A cohort of 210 diabetics with CHD post- PCI was involved. On the basis of the optimal cut-off value for the PIV derived from ROC curve analysis, cases were allocated to either low-PIV (*n* = 104) or high-PIV (*n* = 106) group. The overall study population's mean age was 65.4 ± 8.8 years, with a predominance of male participants (120, 57.1%). As outlined in Table 1, cases in the high-PIV group were remarkably older relative to those in the low-PIV group (67.3 ± 9.1 vs. 63.5 ± 8.4 years, *p* = 0.012), and exhibited a higher prevalence of hypertension (67.6% vs. 49.0%, *p* = 0.008) and multivessel CAD (43.4% vs. 27.2%, *p* = 0.019). No significant differences were observed in other baseline clinical characteristics between the two groups.

**Table 1:** Baseline clinical, laboratory, and angiographic characteristics of patients stratified by pan-immune-inflammatory value (PIV).

Variable	Low PIV (*n* = 104)	High PIV (*n* = 106)	*p*-Value
Age (years)	63.5 ± 8.4	67.3 ± 9.1	0.012
Male sex (*n*, %)	58 (55.8)	62 (58.5)	0.81
BMI (kg/m^2^)	25.6 ± 3.1	26.3 ± 3.4	0.29
Hypertension (*n*, %)	51 (49.0)	72 (67.6)	0.008
Dyslipidemia (*n*, %)	32 (30.6)	43 (40.7)	0.11
Current smoking (*n*, %)	35 (33.7)	39 (36.7)	0.67
HbA1c (%)	7.5 ± 1.1	8.1 ± 1.2	0.034
eGFR (mL/min/1.73 m^2^)	83 ± 19	76 ± 21	0.048
Total cholesterol (mmol/L)	4.52 ± 1.06	4.67 ± 1.11	0.42
LDL-C (mmol/L)	2.61 ± 0.72	2.74 ± 0.81	0.28
HDL-C (mmol/L)	1.06 ± 0.25	1.01 ± 0.28	0.30
Triglycerides (mmol/L)	1.72 ± 0.86	1.83 ± 0.93	0.44
Neutrophils (×10^9^/L)	4.4 ± 1.5	6.9 ± 2.1	<0.001
Monocytes (×10^9^/L)	0.48 ± 0.17	0.72 ± 0.19	<0.001
Lymphocytes (×10^9^/L)	2.21 ± 0.63	1.75 ± 0.58	0.002
Platelets (×10^9^/L)	236 ± 58	282 ± 64	<0.001
PIV (×10^5^)	2.1 ± 0.8	4.6 ± 1.2	<0.001
Multivessel disease (*n*, %)	28 (27.2)	46 (43.4)	0.019
Statin use (*n*, %)	62 (59.2)	66 (62.1)	0.69
Dual antiplatelet therapy (*n*, %)	97 (93.3)	100 (94.3)	0.77

Values’ expression was through mean plus/minus standard deviation or *n* (%).

Patients in the high-PIV group exhibited significantly higher inflammatory cell counts and poorer glycemic and renal profiles compared with the low-PIV group (Table 1). In terms of laboratory parameters, the high-PIV group demonstrated significantly higher neutrophil counts (6.9 ± 2.1 × 10^9^/L vs. 4.4 ± 1.5 × 10^9^/L, *p* < 0.001), monocyte counts (0.72 ± 0.19 × 10^9^/L vs. 0.48 ± 0.17 × 10^9^/L, *p* < 0.001), and platelet counts (282 ± 64 × 10^9^/L vs. 236 ± 58 × 10^9^/L, *p* < 0.001) than the low-PIV group. In contrast, lymphocyte counts were significantly lower in the high-PIV group than in the low-PIV group (1.75 ± 0.58 × 10^9^/L vs. 2.21 ± 0.63 × 10^9^/L, *p* = 0.002).). Lipid profiles and medication use were comparable between the groups.

Collectively, these findings indicate that patients with higher PIV values tended to have more pronounced systemic inflammation, poorer glycemic control, and more complex coronary artery disease. The detailed clinical, laboratory, and procedural characteristics of both groups are summarized in Table 1.

### 3.2 Determination of optimal PIV cut-off

**Figure 1:**
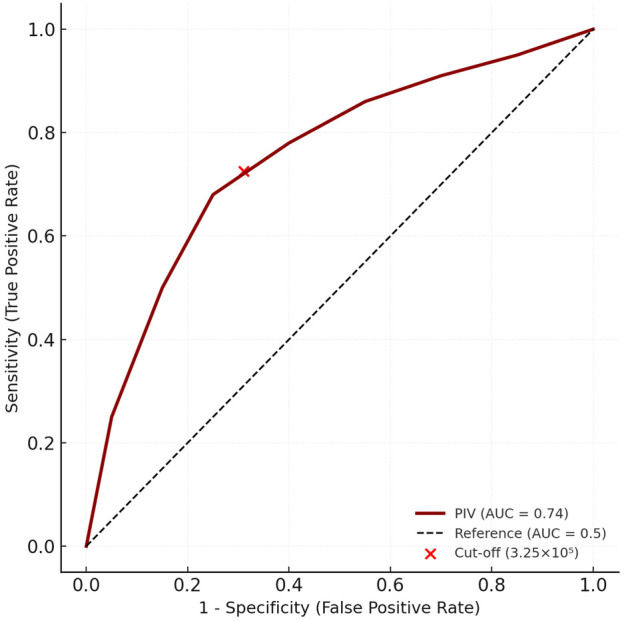
The ROC curve for the PIV in the context of forecasting MACCE post-PCI was generated, wherein the optimal PIV threshold of 3.25 × 10^5^ (denoted by the red dot) yielded a sensitivity of 72.5% and a specificity of 69.3%, resulting in an AUC of 0.74 (95% CI 0.68–0.80), with a *p*-value < 0.001, thereby affirming the robustness of the predictive capacity of PIV. The dashed diagonal line represents the reference line, corresponding to an AUC of 0.5.

Utilizing ROC curve analysis, it was attempted to ascertain PIV's optimal threshold in forecasting MACCE incidence post-PCI. As illuminated in Figure 1, PIV demonstrated a highly robust discriminative capacity, with an AUC of 0.74 (95% CI 0.68–0.80, *p* < 0.001). The optimal cut-off value, derived from the Youden index, was identified at 3.25 × 10^5^, which conferred a sensitivity of 72.5% and a specificity of 69.3% for accurately forecasting MACCE occurrence.

In direct comparison with other inflammation-based indices, PIV exhibited superior diagnostic accuracy (Table 2). PIV demonstrated the highest predictive performance among the evaluated inflammatory indices (Table 2). PIV showed higher predictive performance than NLR and PLR, while the difference compared with SII was not statistically significant. These outcomes reflect the superior predictive accuracy of PIV in forecasting post-PCI cardiovascular and cerebrovascular adverse outcomes in diabetics with CHD. The results not only emphasize the noticeable clinical potential of PIV as a prognostic biomarker. but also reinforce its value in providing detailed, individualized risk stratification.

**Table 2:** Diagnostic performance of PIV and other inflammatory indices in forecasting MACCE.

Index	AUC (95% CI)	Cut-off	Sensitivity (%)	Specificity (%)	*p*-Value vs. PIV
PIV	0.74 (0.68–0.80)	3.25 × 10^5^	72.5	69.3	—
SII	0.69 (0.63–0.76)	1.12 × 10^3^	68.1	63.2	0.087
NLR	0.66 (0.59–0.73)	3.1	64.8	60.7	0.011
PLR	0.64 (0.57–0.71)	137	61.4	58.3	0.006

AUC, area under curve; CI, confidence interval; SII, systemic immune-inflammation index; NLR, neutrophil-to-lymphocyte ratio; PLR, platelet-to-lymphocyte ratio; MACCE, major adverse cardiovascular and cerebrovascular events.

### 3.3 Incidence of adverse cardiovascular and cerebrovascular events

Throughout a median follow-up duration of 24 months (IQR: 18–32 months), 52 MACCEs were totally recorded, corresponding to an overall incidence rate of 24.8%. As outlined in Table 3, a noticeably escalated MACCE incidence was recorded among cases in the high-PIV group relative to those in the low-PIV group (37.7% vs. 11.5%, *p* < 0.001). Further analysis of individual event types revealed that the high-PIV group experienced remarkably greater rates of non-fatal MI (15.1% vs. 5.8%, *p* = 0.021) and ischemic stroke or TIA (9.4% vs. 2.9%, *p* = 0.045). Moreover, the frequency of repeat target-vessel revascularization was notably elevated in the high-PIV group (10.4% vs. 4.8%, *p* = 0.048). Cardiovascular mortality was numerically higher in the high-PIV group but did not reach statistical significance (*p* = 0.18).

**Table 3:** Incidence of MACCE in patients with low and high PIV.

Event	Low PIV (*n* = 104) *n* (%)	High PIV (*n* = 106) *n* (%)	*p*-Value
Total MACCE	12 (11.5)	40 (37.7)	<0.001
Cardiovascular death	3 (2.9)	7 (6.6)	0.18
Non-fatal myocardial infarction	6 (5.8)	16 (15.1)	0.021
Ischemic stroke/TIA	3 (2.9)	10 (9.4)	0.045
Repeat revascularization	5 (4.8)	11 (10.4)	0.048

Values’ expression was in form of number (percentage) of cases. MACCE, major adverse cardiovascular and cerebrovascular events; TIA, transient ischemic attack.

Patients with high PIV had a significantly higher incidence of MACCE of both cardiovascular and cerebrovascular complications post-PCI in diabetics with CAD. This robust link reflects the potential of PIV as an invaluable, easily accessible prognostic biomarker.

### 3.4 KM survival analysis

**Figure 2:**
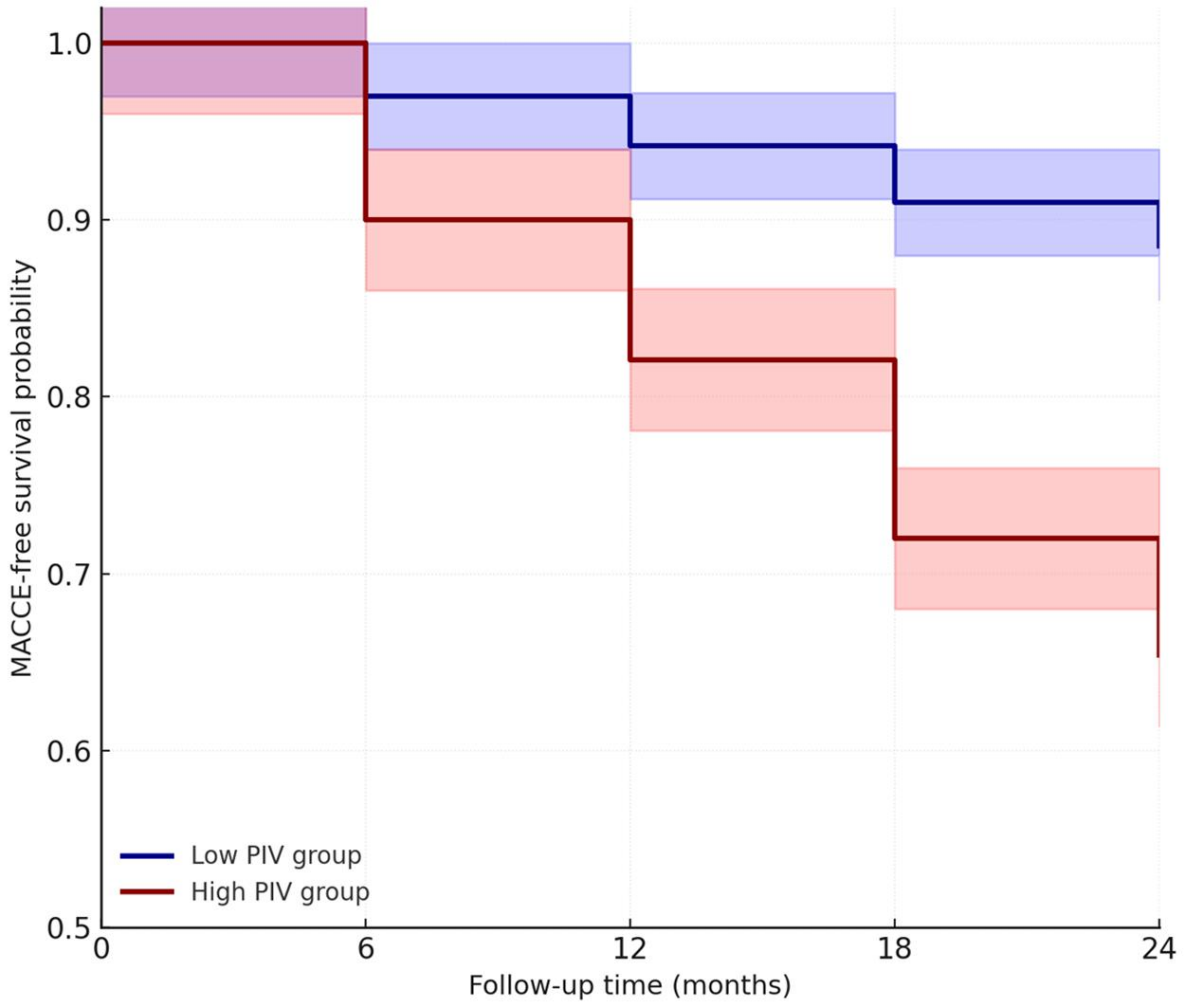
The Kaplan–Meier survival curves, stratified by PIV group, illustrate the cumulative probability of remaining free from MACCE over a 24-month follow-up period. These curves reveal that cases in the high-PIV group (depicted by the red line) exhibited a noticeably attenuated MACCE-free survival relative to their counterparts in the low-PIV group (represented by the blue line), with the difference yielding statistical significance as indicated by a log-rank *p*-value of less than 0.001. The shaded regions surrounding the curves denote the 95% CIs.

KM curves were drawn to compare the cumulative incidence of MACCE between the two principal groups. As depicted in Figure 2, cases with elevated pre-procedural PIVs demonstrated a remarkably attenuated MACCE-free survival rate relative to those with lower PIVs (log-rank *p* < 0.001). Kaplan–Meier analysis showed significantly lower MACCE-free survival in the high-PIV group throughout follow-up (Figure 2), with survival rates of 88.5% and 65.4%, respectively. The separation between the survival curves became evident as early as six months post-PCI, with the gap progressively widening throughout the remainder of the observation period. These outcomes unequivocally demonstrate that a higher pre-procedural PIV is intimately linked to a noticeably escalated risk of long-term cardiovascular and cerebrovascular complications, reinforcing its value as a robust and easily measurable inflammation-based biomarker for risk stratification in diabetics post-PCI.

### 3.5 Univariate and multivariate CR analyses

Utilizing CPHR models, identification of independent prognostic factors for the occurrence of MACCE was undertaken particularly during the follow-up period. In the univariate analysis, remarkable linkages of MACCE with several variables was noted, comprising age, hypertension, multivessel CAD, HbA1c, eGFR, and elevated PIV (Table 4). Notably, high PIV emerged as one of the most potent predictors, with a HR of 3.56 (95% CI: 2.01–6.30, *p* < 0.001) relative to the low-PIV group. Variables exhibiting a *p*-value falling below 0.10 in the univariate analysis were thereafter involved in the multivariate CR model. After adjustment for the above-described potential confounders, high PIV remained a robust independent predictor of MACCE, with an adjusted HR of 2.87 (95% CI: 1.55–5.32, *p* = 0.001). In addition to PIV, the presence of multivessel CAD (HR: 1.82, 95% CI: 1.02–3.26, *p* = 0.042) and higher HbA1c levels (per 1% increase; HR: 1.21, 95% CI: 1.03–1.43, *p* = 0.023) also retained their significance in the fully adjusted model. In contrast, variables, consisting of age, hypertension, and eGFR, lost statistical significance following adjustment, reflecting their lesser impact in the context of escalated PIV and other clinical factors. These outcomes emphasize that a higher pre-procedural PIV provides meaningful prognostic insight, reflecting an incremental risk stratification benefit beyond conventional clinical and biochemical markers, and remains independently linked to a noticeably escalated risk of long-term cardiovascular and cerebrovascular adverse events post-PCI in diabetics with CHD.

**Table 4:** Univariate and multivariate Cox regression analyses of MACCE's predictors.

Variable	Univariate HR (95% CI)	*p*-Value	Multivariate HR (95% CI)	*p*-Value
Age (per 10 years)	1.32 (1.01–1.72)	0.041	1.18 (0.89–1.56)	0.25
Male sex	1.09 (0.63–1.88)	0.76	–	–
Hypertension	1.74 (1.02–2.96)	0.042	1.39 (0.80–2.41)	0.24
Multivessel disease	2.01 (1.17–3.45)	0.011	1.82 (1.02–3.26)	0.042
HbA1c (per 1% increase)	1.25 (1.08–1.44)	0.003	1.21 (1.03–1.43)	0.023
eGFR (per 10 mL/min/1.73 m^2^ decrease)	1.19 (1.02–1.40)	0.029	1.11 (0.94–1.32)	0.22
LDL-C (per 1 mmol/L)	1.09 (0.87–1.37)	0.45	–	–
High PIV (vs. low PIV)	3.56 (2.01–6.30)	<0.001	2.87 (1.55–5.32)	0.001

MACCE, major adverse cardiovascular and cerebrovascular events; HR, hazard ratio; CI, confidence interval; eGFR, estimated glomerular filtration rate; LDL-C, low-density lipoprotein cholesterol; PIV, pan-immune-inflammatory value. Variables with *p* < 0.10 in univariate analysis were entered into the multivariate model.

### 3.6 Subgroup and sensitivity analyses

**Figure 3:**
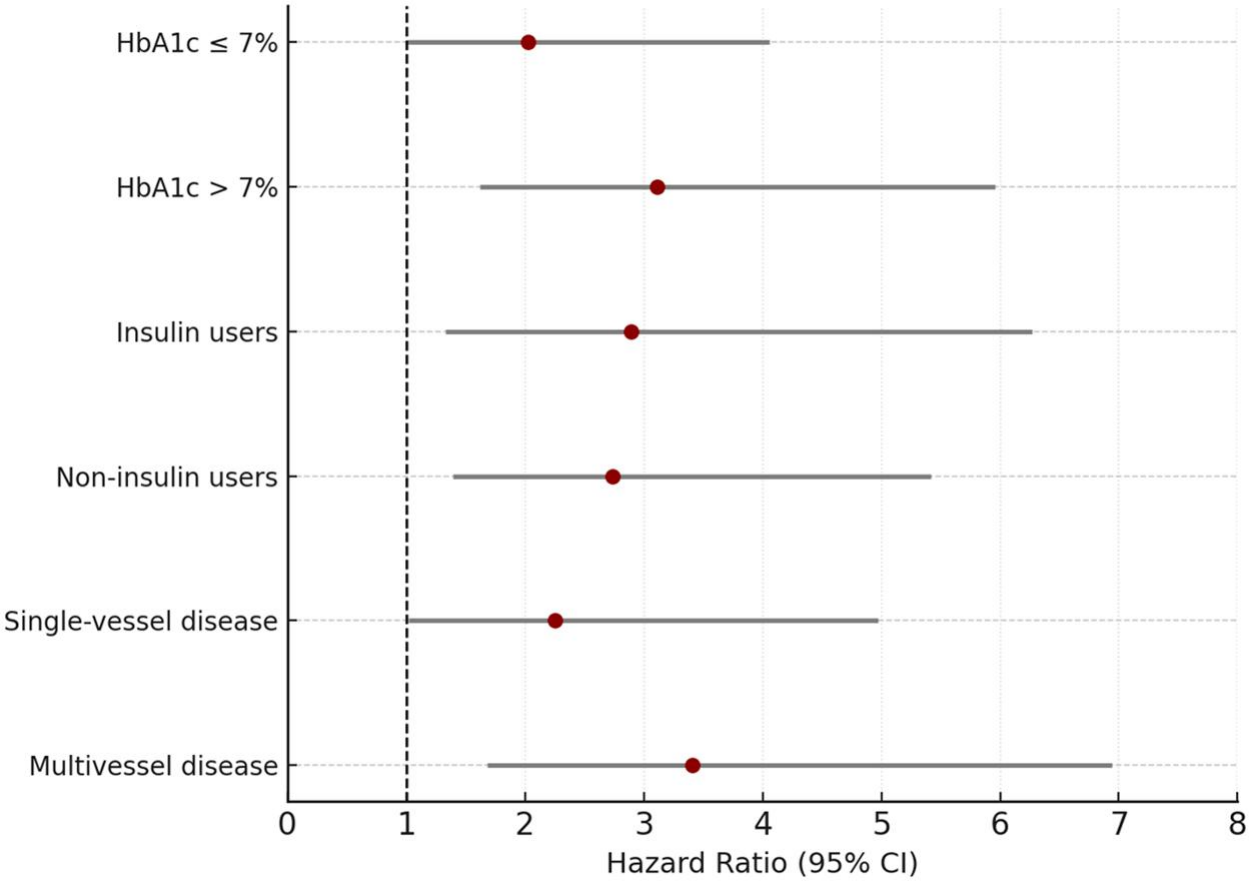
Illustrating the outcomes of subgroup analyses examining the link of high PIV with the occurrence of MACCE through the forest plot. Each point on the plot represents the HR with 95% CIs for the comparable analysis of high vs. low PIV across various clinical subgroups. The analysis uncovered that an escalated PIV could consistently forecast an elevated risk of MACCE in all examined subgroups, involving those categorized by glycemic control (HbA1c ≤7% vs. >7%), insulin usage, and the CAD extent. The dashed vertical line signifies the null value (HR = 1.0).

To thoroughly examine the consistency of the PIV as a prognostic marker across various clinical contexts, stratified CR analyses were implemented for different subgroups, the results of which are outlined in Figure 3. The link of escalated PIV with the incidence of MACCE remained statistically significant in each of the predefined subgroups. Specifically, in cases exhibiting poor glycemic control (defined as HbA1c > 7%), high PIV was robustly linked to an escalated risk of MACCE (HR = 3.11, 95% CI: 1.62–5.96, *p* < 0.001), whereas, in those with better glycemic control (HbA1c ≤ 7%), this link, No significant interaction effects were observed across subgroup analyses.

Furthermore, high PIV's prognostic significance remained evident in both subgroups based on insulin therapy, with HRs of 2.89 (95% CI: 1.33–6.27, *p* = 0.007) in insulin users and 2.74 (95% CI: 1.39–5.42, *p* = 0.004) in non-insulin users, and the absence of significant interaction was noteworthy between insulin therapy status and the impact of PIV (p for interaction = 0.77). Moreover, when the cohort was stratified by the CAD complexity, PIV continued to be a notable predictor of adverse outcomes in both single-vessel (HR = 2.25, 95% CI: 1.02–4.97, *p* = 0.045) and multivessel disease (HR = 3.41, 95% CI: 1.68–6.94, *p* = 0.001), without remarkable interaction particularly between lesion complexity and PIV (p for interaction = 0.33). Consequently, these outcomes affirm the robustness and consistency of PIV as a reliable independent predictor of long-term MACCE post-PCI, reflecting that its prognostic significance persists across variations in glycemic control, insulin therapy usage, and the anatomical severity of coronary disease.

### 3.7 Comparative predictive performance

To further figure out the relative prognostic efficacy of the PIV vs. other inflammation-based indices, a comprehensive evaluation was implemented, integrating both the AUC and HRs for MACCE across four indices (PIV, SII, NLR, and PLR). As documented in Table 5, PIV emerged as the most robust discriminator, yielding an AUC of 0.74 (95% CI: 0.68–0.80), surpassing the discriminatory power of SII (AUC = 0.69, 95% CI: 0.63–0.76), NLR (AUC = 0.66, 95% CI: 0.59–0.73), and PLR (AUC = 0.64, 95% CI: 0.57–0.71). When subjected to multivariate CR analysis, the escalated PIV was found to exhibit the most remarkable and independent link with MACCE, accompanying by a HR of 2.87 (95% CI: 1.55–5.32, *p* = 0.001). Although SII and NLR were also independently linked to the escalated incidence of MACCE (HR = 1.79, 95% CI: 1.11–2.87, *p* = 0.017 and HR = 1.66, 95% CI: 1.12–2.47, *p* = 0.011, respectively), PLR lost its remarkable significance post-adjustment for confounding variables. PIV demonstrated the highest predictive performance among the evaluated inflammatory indices, but also retains superior prognostic capability, rendering its potential as a more reliable and clinically relevant systemic inflammation biomarker for post-PCI risk stratification in diabetics with CHD.

**Table 5:** Comparative predictive performance of inflammatory indices for MACCE.

Index	AUC (95% CI)	HR (95% CI)	*p*-Value
PIV	0.74 (0.68–0.80)	2.87 (1.55–5.32)	0.001
SII	0.69 (0.63–0.76)	1.79 (1.10–2.91)	0.019
NLR	0.66 (0.59–0.73)	1.66 (1.02–2.70)	0.041
PLR	0.64 (0.57–0.71)	1.28 (0.79–2.09)	0.30

AUC, area under curve; HR, hazard ratio; CI, confidence interval; PIV, pan-immune-inflammatory value; SII, systemic immune-inflammation index; NLR, neutrophil-to-lymphocyte ratio; PLR, platelet-to-lymphocyte ratio; MACCE, major adverse cardiovascular and cerebrovascular events.

## 4 Discussion

This study evaluated the prognostic significance of PIVin forecasting MACCE in diabetics with CHD post-PCI. The principal findings uncovered that an escalated pre-procedural PIV was remarkably linked to an elevated MACCE incidence, involving MI, ischemic stroke, and the need for repeat revascularization. Notably, even after rigorous multivariate adjustments, PIV retained its status as an independent predictor of adverse outcomes, while simultaneously demonstrating superior prognostic accuracy relative to other established inflammatory markers, involving SII, NLR, and PLR. These outcomes position PIV as a simple, cost-effective, and highly robust biomarker for long-term risk stratification in diabetics post-PCI.

Chronic inflammation is recognized as a central contributor to adverse cardiovascular outcomes in diabetic patients with CHD. In the present study, elevated PIV was independently associated with increased MACCE risk after PCI, supporting the growing evidence that inflammation-based biomarkers may improve cardiovascular risk stratification in high-risk diabetic populations (11, 12).Even after technically successful PCI, diabetic patients often experience persistent inflammatory activation, leading to restenosis, thrombosis, or progressive vascular injury (13). Therefore, identifying reliable inflammatory markers that capture this systemic immune imbalance is crucial for improving prognostic assessment and guiding personalized therapy.

Compared with conventional inflammatory indices, PIV demonstrated superior prognostic performance, likely because it incorporates multiple immune and inflammatory cell components into a single composite marker. The prognostic value of PIV may be attributable to its integration of multiple components of the inflammatory and immune response. Neutrophils and monocytes contribute to vascular inflammation and endothelial injury (14), whereas platelets promote thrombo-inflammatory processes and vascular complications (15). In contrast, lymphocytes play an important role in immune regulation, and reduced lymphocyte counts have been associated with adverse cardiovascular outcomes (16). By combining these cellular components into a single index, PIV may provide a more comprehensive assessment of systemic inflammatory and immune status than individual hematological parameters or simpler inflammatory ratios. The combination of high neutrophil, monocyte, and platelet counts with low lymphocyte levels hallmarks of a high PIV captures this shift toward a proinflammatory, prothrombotic state. In our study, patients with high PIV values displayed higher neutrophil, monocyte, and platelet counts, lower lymphocyte levels, and greater prevalence of multivessel disease and poor glycemic control, confirming that PIV mirrors systemic inflammatory load in diabetic CHD patients.

ROC analysis in this study demonstrated that PIV provided better discriminative ability for predicting MACCE than traditional markers. The AUC for PIV was 0.74, significantly higher than for NLR (0.66) and PLR (0.64), and slightly better than for SII (0.69). This suggests that while all indices reflect systemic inflammation, PIV offers a more comprehensive evaluation of immune activation. Similar findings have been reported in recent studies. Aydin et al. observed that PIV predicted no-reflow and mortality after PCI in patients with acute myocardial infarction, outperforming other hematologic indices (17). Likewise, Fan et al. found that PIV was independently associated with adverse events in acute coronary syndrome and had stronger predictive accuracy than SII and NLR (18). Our findings extend this evidence by demonstrating the prognostic significance of PIV specifically in diabetic CHD patients, who constitute a subgroup with distinct pathophysiological characteristics and higher event rates.

The Kaplan–Meier survival analysis confirmed the long-term predictive value of PIV. High-PIV patients had significantly lower MACCE-free survival at both 12 and 24 months, with early divergence between survival curves within the first six months post-PCI. This pattern suggests that PIV captures both early inflammatory vulnerability and chronic vascular instability. The consistent predictive performance of PIV across subgroups regardless of glycemic control, insulin therapy, or disease extent supports its robustness. Notably, the association between PIV and MACCE was strongest among patients with HbA1c > 7%, highlighting the interaction between hyperglycemia and inflammation. Hyperglycemia induces monocyte adhesion, reactive oxygen species generation, and platelet activation, amplifying inflammatory signaling (19). Therefore, PIV may act as a surrogate measure of cumulative inflammatory stress resulting from poor glycemic control, explaining its pronounced prognostic value in uncontrolled diabetic populations.

In multivariate CR, high PIV remained an independent predictor of MACCE even after adjusting for age, hypertension, HbA1c, and renal function (adjusted HR = 2.87, 95% CI: 1.55–5.32, *p* = 0.001). This finding indicates that the prognostic effect of PIV extends beyond traditional cardiovascular risk factors. Multivessel disease and elevated HbA1c were also independently associated with events, consistent with prior reports linking poor glycemic control and coronary complexity to worse outcomes after PCI (20). The persistence of PIV as a strong predictor even in adjusted models underscores its clinical value as a composite inflammation index integrating both vascular and metabolic risk elements.

Comparative performance analysis further strengthened the relevance of PIV. Among the inflammatory indices evaluated, PIV not only exhibited the highest AUC but also yielded the strongest hazard ratio for predicting MACCE. This superior predictive ability likely reflects the integration of monocytes and platelets into the PIV formula—components not captured by simpler indices like NLR or PLR. Monocytes contribute directly to atherosclerotic plaque formation through macrophage differentiation and foam cell generation, while activated platelets amplify inflammatory signaling by releasing CD40 ligand and thromboxane A_2_ (21). The inclusion of these variables provides PIV with a broader representation of the inflammatory milieu than indices based on leukocytes alone.

The present findings suggest that PIV may have practical value in clinical risk stratification for diabetic patients undergoing PCI. Because PIV is derived from routine complete blood count parameters, it can be rapidly calculated without additional cost or specialized testing. Integration of PIV into pre-procedural assessment models may help identify patients at elevated inflammatory and thrombotic risk who require closer surveillance after PCI. In clinical practice, patients with high PIV levels may benefit from intensified secondary prevention strategies, including stricter glycemic control, optimization of lipid-lowering and antiplatelet therapy, and more frequent cardiovascular follow-up. PIV may also assist clinicians in identifying individuals with persistent residual inflammatory risk despite standard medical therapy. Furthermore, combining PIV with established clinical and angiographic risk factors could improve individualized prognostic assessment beyond traditional cardiovascular markers alone.

The incorporation of inflammation-based biomarkers such as PIV into routine PCI care pathways may contribute to more personalized management approaches in diabetic CHD patients. However, before widespread clinical implementation, prospective multicenter studies are needed to validate optimal cut-off values, evaluate longitudinal changes in PIV, and determine whether PIV-guided management strategies can improve long-term cardiovascular outcomes.

Additionally, serial measurement of PIV might allow dynamic monitoring of inflammation during post-PCI recovery, offering opportunities for early intervention when inflammatory activity persists. Incorporating PIV into predictive models could refine existing risk stratification systems, complementing traditional clinical and biochemical markers.

The prognostic relevance of inflammation-based biomarkers extends beyond coronary artery disease and diabetes mellitus to other complex vascular disorders characterized by chronic immune and inflammatory activation. Recent evidence suggests that inflammatory biomarkers may also have prognostic significance in rare cerebrovascular diseases such as Moyamoya disease, a progressive occlusive cerebrovascular disorder associated with vascular remodeling and ischemic complications. Previous clinicopathological investigations have highlighted the contribution of inflammatory and immune-mediated mechanisms to endothelial dysfunction, abnormal vascular proliferation, and disease progression in Moyamoya disease. In this context, biomarkers reflecting systemic inflammatory burden may provide additional insight into disease severity, vascular instability, and long-term clinical outcomes. For example, Pop et al. emphasized the importance of inflammatory and vascular pathological mechanisms in Moyamoya disease and discussed the potential value of inflammatory evaluation in understanding disease progression and cerebrovascular injury. Similar to coronary artery disease, persistent inflammation may contribute to vascular stenosis, ischemic events, and impaired vascular repair processes in Moyamoya patients. These observations further support the broader clinical relevance of composite inflammatory biomarkers such as PIV in complex cardiovascular and cerebrovascular disorders. Future studies may investigate whether PIV and related inflammatory indices could also contribute to prognostic assessment and individualized management strategies in patients with rare inflammatory vascular diseases (22). Landmark trials such as CANTOS and COLCOT have demonstrated that modulating inflammatory pathways with canakinumab or colchicine significantly lowers recurrent ischemic events, independent of lipid levels (23, 24). Within this context, PIV could serve as a pragmatic surrogate marker to identify patients with residual inflammatory risk who might benefit most from such therapies. This approach is particularly relevant for diabetic CHD patients, who exhibit a persistent inflammatory phenotype despite optimal medical management.

The strong association between elevated PIV and adverse outcomes observed in this study suggests that systemic inflammatory burden may contribute to long-term cardiovascular and cerebrovascular complications following PCI (25). Moreover, lymphopenia, a key component of high PIV, is a marker of systemic stress and immune exhaustion, which have been associated with poor cardiovascular prognosis (26). Therefore, PIV represents a cumulative measure of systemic immune imbalance rather than a single inflammatory pathway, explaining its strong and independent prognostic impact.

Several limitations of this study should be acknowledged. First, this was a retrospective single-center study with a relatively moderate sample size, which may introduce selection bias and limit the generalizability of the findings to broader populations and different clinical settings. Second, although consecutive patients were included, the observational study design does not allow establishment of a causal relationship between elevated PIV and adverse cardiovascular outcomes.

Third, PIV was assessed only at a single pre-procedural time point. Dynamic changes in inflammatory status during follow-up were not evaluated, and serial PIV measurements may provide additional prognostic information regarding persistent or residual inflammatory risk after PCI. Fourth, although multivariate analyses adjusted for several established cardiovascular risk factors, residual confounding cannot be completely excluded. Potentially relevant variables such as duration of diabetes, medication adherence, inflammatory comorbidities, socioeconomic factors, dietary status, and lifestyle characteristics were not comprehensively assessed.

In addition, the study did not evaluate whether incorporation of PIV into existing clinical risk prediction models improves prognostic discrimination or clinical decision-making. Therefore, large prospective multicenter studies with longitudinal inflammatory assessment are needed to validate these findings, establish standardized PIV cut-off values, and determine the clinical utility of PIV-guided management strategies in diabetic patients undergoing PCI.

In this study demonstrates that elevated PIV is an independent and superior predictor of adverse cardiovascular and cerebrovascular outcomes after PCI in diabetic patients with CHD. By integrating neutrophil, monocyte, platelet, and lymphocyte counts, PIV integrates neutrophil, monocyte, platelet, and lymphocyte counts and may reflect the overall systemic inflammatory and immune status of patients undergoing PCI. The observed association between elevated PIV and adverse clinical outcomes suggests that PIV may serve as a useful prognostic biomarker for identifying patients at increased risk of MACCE. However, the present study was not designed to establish causal mechanisms linking PIV to atherosclerotic progression or post-PCI complications. Its simplicity, availability, and strong prognostic performance make it a promising biomarker for risk stratification and potentially for guiding anti-inflammatory therapeutic strategies in this high-risk population.

Another limitation is that established inflammatory biomarkers such as high-sensitivity C-reactive protein (hs-CRP) and interleukin-6 (IL-6) were not routinely available in the present cohort and therefore could not be included in the comparative analyses. Although PIV demonstrated superior predictive performance compared with other blood cell-derived inflammatory indices, direct comparisons with hs-CRP and IL-6 were not possible. Future prospective studies incorporating these biomarkers are warranted to further define the relative prognostic value of PIV in patients undergoing.

## 5 Conclusion

In conclusion, elevated pre-procedural PIV was independently associated with an increased risk of major adverse cardiovascular and cerebrovascular events in diabetic patients with coronary heart disease undergoing PCI. PIV demonstrated superior predictive performance compared with other blood cell-derived inflammatory indices and remained prognostically significant across clinically relevant subgroups. Given its availability from routine blood tests, PIV may represent a practical tool for risk stratification in this high-risk population. Future prospective multicenter studies are needed to validate these findings and determine the clinical utility of PIV-guided management strategies.

## Data Availability

The original contributions presented in the study are included in the article/Supplementary Material, further inquiries can be directed to the corresponding author/s.
